# Molecular analysis of hemoglobinopathies in a large ethnic Hakka population in southern China

**DOI:** 10.1097/MD.0000000000013034

**Published:** 2018-11-09

**Authors:** Pingsen Zhao, Heming Wu, Ruiqiang Weng

**Affiliations:** a Clinical Core Laboratory; b Center for Precision Medicine, Meizhou People's Hospital (Huangtang Hospital), Meizhou Academy of Medical Sciences, Meizhou Hospital Affiliated to Sun Yat-sen University; c Guangdong Provincial Engineering and Technology Research Center for Molecular Diagnostics of Cardiovascular Diseases; d Meizhou Municipal Engineering and Technology Research Center for Molecular Diagnostics of Cardiovascular Diseases; e Meizhou Municipal Engineering and Technology Research Center for Molecular Diagnostics of Major Genetic Disorders; f Prenatal Diagnosis Center, Meizhou People's Hospital (Huangtang Hospital), Meizhou Academy of Medical Sciences, Meizhou Hospital Affiliated to Sun Yat-sen University, Meizhou, P. R. China.

**Keywords:** hemoglobinopathies, Hakka, molecular epidemiological survey, southern China, thalassemia

## Abstract

Thalassemia is an inherited autosomal recessive disorder with microcytic hypochromic anemia resulting from reduced or absent synthesis of 1 or more of the globin chains of hemoglobin. This study provided the insight into prevalence and molecular characterization of thalassemia in Hakka population. 14,524 unrelated subjects were included in our study from January 2015 to November 2017. All the subjects were detected by hematological analysis, hemoglobin electrophoresis analysis, and molecular diagnosis (gap-polymerase chain reaction and flow-through hybridization technology). Data analysis was used to compare allele frequencies between the Hakka populations. Seven thousand four hundred twenty-two cases of microcytosis were found. The percentage of microcytosis in Meizhou, Ganzhou, and Heyuan was 50.91% (6738/13,236), 51.27% (445/868), and 56.90% (239/420), respectively. A total of 5516 mutant chromosomes were identified, including 3775 α-thalassemia and 1741 β-thalassemia. --^SEA^/αα was the most common α-thalassemia genotype, followed by -α^3.7^/αα and -α^4.2^/αα, accounted for 84.92% of α-thalassemia genotypes. Twelve kinds of mutations and 26 genotypes in β-thalassemia were found. IVS-II-654(C→T), CD41-42(-TCTT), −28(A→G), and CD17(A→T) alleles accounted for 92.65% of these mutations. IVS-II-654/N, CD41-42/N, -28/N, CD17/N genotypes accounted for 91.53% of β-thalassemia genotypes. 27 fetuses with at-risk pregnancies were subjected to prenatal diagnosis. Five fetuses were Bart's hydrops syndrome and 2 fetuses with β-thalassemia major. There were some differences in molecular characterization of thalassemia among Hakka people in different areas of southern China. Our results enriched the related information of thalassemia in the region, which provided valuable references for the prevention and control of thalassemia.

## 1 Introduction

Thalassemia is an inherited autosomal recessive disease with microcytic hypochromic anemia resulting from reduced or absent synthesis of 1 or more of the globin chains of hemoglobin. Clinical phenotype of patients with thalassemia varies from almost asymptomatic to a lethal hemolytic anemia.^[1–3]^ Thalassemia is the most common monogenic disorder in the world and especially prevalent in Mediterranean countries, Southeast Asia, Africa, Middle East, and in the Indian subcontinent. There are 2 main types of thalassemia, α and β.^[4,5]^

Previous studies have shown that there was a high-frequency of thalassemia in population of southern China,^[6–9]^ particularly in the 3 provinces of Guangdong,^[10,11]^ Guangxi,^[12]^ and Hainan.^[13,14]^ Meizhou is a city situated at the northeast of Guangdong province at the junction of Fujian, Guangdong, and Jiangxi provinces, with an area of 15,876 km^2^ and a population of 5.44 million. The vast majority of the residents living in this area are Hakka peoples. Hakka is an intriguing Han Chinese population that mainly inhabit in southern China who migrated to south originally from northern China.^[15]^

There is no ideal method for treatment of severe thalassemia, and it is a blood disease resulting in fatality or crippling.^[16]^ Large-scale population genetic screening, genetic counseling, and prenatal diagnosis to avoid affected births are the best choice. In the present study, we perform a large-scale survey of thalassemia in 14,524 subjects to analyze the prevalence and molecular characteristics of thalassemia in Hakka population. It will provide valuable reference for the prevention and control of thalassemia in this area.

## 2 Materials and methods

### 2.1 Study population

**Figure 1:**
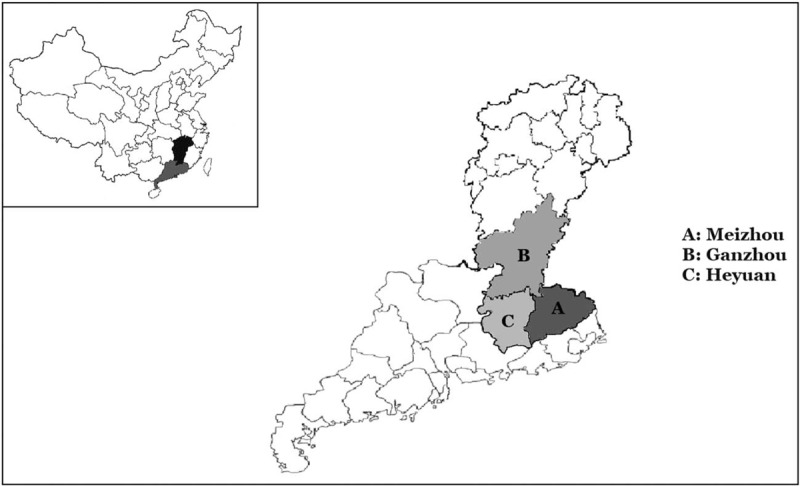
The geographical position of the 3 study regions including Meizhou region (A), Ganzhou region (B), and Heyuan region (C).

**Figure 2:**
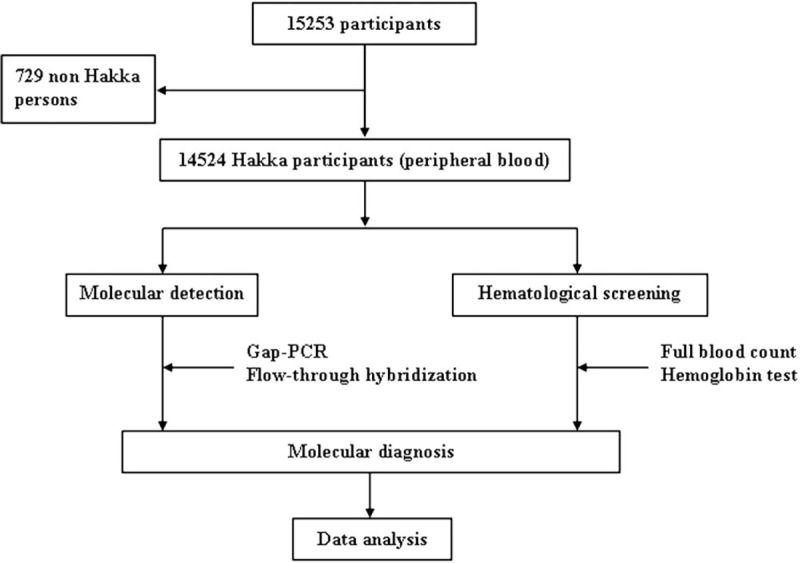
Diagnostic flowchart for the detection of thalassemia in this study.

Fifteen thousand two hundred fifty-three unrelated subjects who visited our hospital between January 2015 and November 2017 were collected for this study, and 95.22% (14,524/15,253) of them were of Hakka descent. Figure 1 showed the location of the 3 regions of study in Meizhou (A) (13,236 subjects), Ganzhou (B) (868 subjects), and Heyuan (C) (420 subjects). These subjects visited Meizhou People's Hospital (Huangtang Hospital), Meizhou Hospital Affiliated to Sun Yat-sen University for routine examination. Diagnostic flowchart for the detection of thalassemia in this study showed in Figure 2. The study was performed in accordance with the Declaration of Helsinki and was approved by the Ethics Committee of the Meizhou People's Hospital (Huangtang Hospital), Meizhou Hospital Affiliated to Sun Yat-sen University.

### 2.2 Hematological analysis and hemoglobin electrophoresis analysis

Samples were obtained via venipuncture of an antecubital vein using Ethylenediaminetetraacetic acid (EDTA) anticoagulant tube collection, 2 mL peripheral blood was collected for relative detection. Erythrocyte correlation indices were determined followed the standard laboratory procedures provided by Sysmex XE-2100 blood analyzer (Sysmex, Inc., Japan). Subjects with low mean corpuscular volume (MCV) values (<82 fl) were considered possibly thalassemia carriers.

Hemoglobin electrophoresis analysis was determined according to standard laboratory procedures by Sebia capillary electrophoresis system (Sebia, Inc., France). Subjects with low HbA_2_ (<2.5%) were considered possibly α-thalassemia carriers, with high HbA_2_ (>3.5%) were considered possibly β-thalassemia carriers, respectively.

### 2.3 DNA extraction and genotyping

Genomic DNAs of subjects were extracted from peripheral blood leukocytes by QIAamp DNA Blood Mini Kit (Qiagen, Germany) according to the manufacturer's instructions. DNA concentration and purity were quantified using Nanodrop 2000TM Spectrophotometer (ThermoFisher Scientific, Waltham, MA) at the wavelength of 260 nm, and only good quality DNA (A260/280 >1.7) were stored at −80°C until analyzed.

Gap-polymerase chain reaction (gap-PCR) and flow-through hybridization technology (Hybribio Limited, Chaozhou, China) were used to detect α-thalassemia mutations, including deletion mutations of --SEA, -α^3.7^ and -α^4.2^ and non-deletion mutations of Hb Constant Spring (α^CS^α) (CD142, TAA→CAA), Hb Quong Sze (α^QS^α) (CD125, CTG→CCG), and Hb Westmead (α^WS^α) (CD122, CAC→CAG). Polymerase chain reaction for detection α-thalassemia mutations was performed according to the following protocol: denaturation at 95°C for 15 minutes, and then 35 cycles of amplification, with 40 seconds at 98°C for denaturation, 1 minute and 10 seconds at 64°C for annealing, and 2 minutes and 30 seconds at 72°C for elongation. Mutations in the β-globin gene were detected by polymerase chain reaction (PCR) and flow-through hybridization technology (Hybribio Limited, China), specifically detection of the following 16 common non-deletion β-globin gene mutations: CD41-42(-TCTT), CD43(G→T), IVS-II-654(C→T), CD17(A→T), CD14-15(+G), -28(A→G), -29(A→G), CD71-72(+A), CD26(G→A), IVS-I-1(G→T), IVS-I-1(G→A), CD27- 28(+C), IVS-I-5(G→C), Cap+40-43(-AAAC), initiation codon (T→G), and CD31(-C). Polymerase chain reaction for detection β-thalassemia mutations was performed according to the following protocol: 37°C for 5 minutes, initial denaturation at 94°C for 4 minutes, and then 40 cycles of amplification, with 30 seconds at 94°C for denaturation, 30 seconds at 55°C for annealing, and 30 seconds at 72°C for elongation. Flow-through hybridization was operated according to the manufacturer's instructions.

### 2.4 Multiplex ligation-dependent probe amplification analysis (MLPA) for rare types of deletion-thalassemia

Samples of suspected rare deletion types were used to detect rare types of deletion-mutational thalassemia by MLPA according to the manufacturer's instructions. Ligation and amplification were carried out on a thermal cycler (Bio-RAD, CA). Amplified fragments were separated by capillary electrophoresis on an ABI 3500 Genetic Analyzer (Applied Biosystems, CA). The area under the peak for each amplified fragment was measured and normalized in comparison with the peak areas of normal control individuals using GeneMarker software v.1.8 (Soft-Genetics, PA). Threshold ratios for deletion and duplication were set at <.6 and >1.3, respectively.

### 2.5 Molecular prenatal diagnosis of α- and β-thalassemia

If both parents are carriers with the same type of alpha- or beta-thalassemia, there is 25% probability for fetus suffering from thalassemia major. Molecular prenatal diagnosis of alpha- and beta-thalassemia was carried out in parents who carried same thalassemia to prevent the births of children with thalassemia major. Fetal sampling was performed in 3 ways:

(1) Chorionic villi sampling (CVS) was conducted at 10 to 12 weeks of gestation;
(2) amniotic fluid (10 mL) was collected at 15 to 22 weeks;
(3) cord blood (1.0–2.0 mL) was sampled at 18 to 28 weeks.^[17,18]^

All procedures were carried out under ultrasonography guidance. At the same time, 2 mL maternal peripheral blood was sampled for short tandem repeats (STR) analysis using Sanger sequencing to identify maternal cell contamination (MCC).^[19,20]^

### 2.6 Statistical analysis

SPSS statistical software version 19.0 was used for data analysis. The data were expressed as the means ± SD. Descriptive analysis was used to compare allele frequencies among the Hakka populations. A value of *P* <.05 was considered as statistically significant.

## 3 Results

### 3.1 Population characteristics

A total of 15,253 participants were subjected, screened out 729 non Hakka persons, 14,524 Hakka persons were analyzed from Meizhou, Ganzhou, and Heyuan, including 5955 males and 8569 females (1:1.439). The ages of these subjects ranged from 12 days to 99 years old and about 91.13% were Meizhou Hakka natives.

### 3.2 Prevalence and mutation spectrum of α- and β-thalassemia

Seven thousand four hundred twenty-two cases of microcytosis (MCV <82fL) were found. The percentage of microcytosis in Meizhou, Ganzhou, and Heyuan was 50.91% (6,738/13,236), 51.27% (445/868), and 56.90% (239/420), respectively. Results of 14,524 cases of hematological screening were shown in Table 1.

**Table 1:**
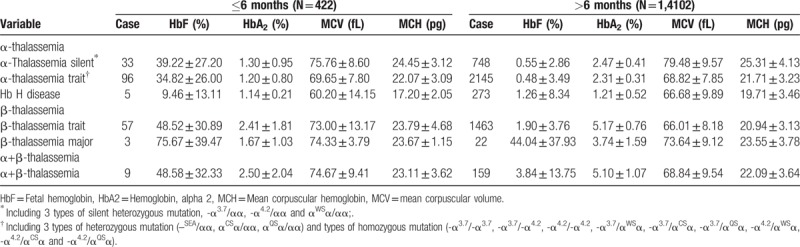
Results of 14524 cases of hematological screening.

All Hakka participants were analyzed gene chip for the 3 known α-thalassemia deletions, 3 α-thalassemia mutations and 16 known common β-thalassemia mutations in Chinese. A total of 5516 mutant chromosomes were identified, including 3775 α-thalassemia and 1741 β-thalassemia. The results of α- and β-thalassaemia prevalence among these regions in Hakka were shown in Table 2. The results of α- and β-thalassaemia alleles and their distribution among these regions in Hakka were shown in Table 3.

**Table 2:**
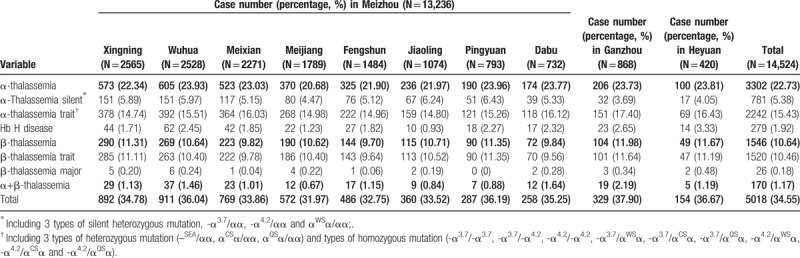
Prevalence of α- and β-thalassaemia among Hakkas in these regions.

**Table 3:**
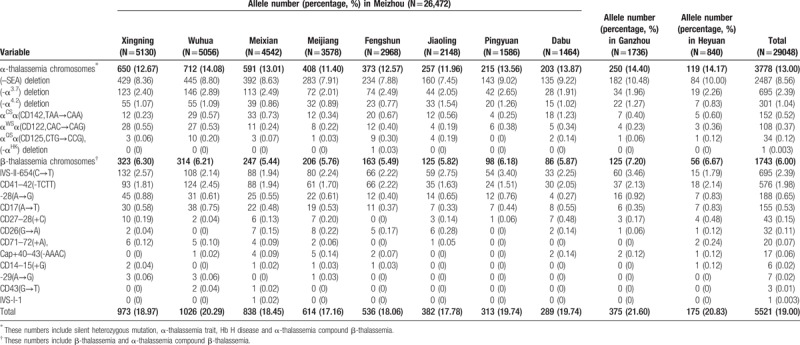
α- and β-thalassaemia alleles and their distribution among Hakkas in these regions.

As the Table 4  shown, --^SEA^/αα was the most common α-thalassemia genotype, followed by -α^3.7^/αα and -α^4.2^/αα, accounted for 84.92% of α-thalassemia genotypes. Twelve kinds of mutations and 26 genotypes in β-thalassemia were found in the molecular survey. IVS-II-654(C→T), CD41-42(-TCTT), -28(A→G), and CD17(A→T) alleles accounted for 92.65% of these mutations. IVS-II-654/N, CD41- 42/N, -28/N, CD17/N genotypes accounted for 91.53% of β-thalassemia genotypes.

**Table 4:**
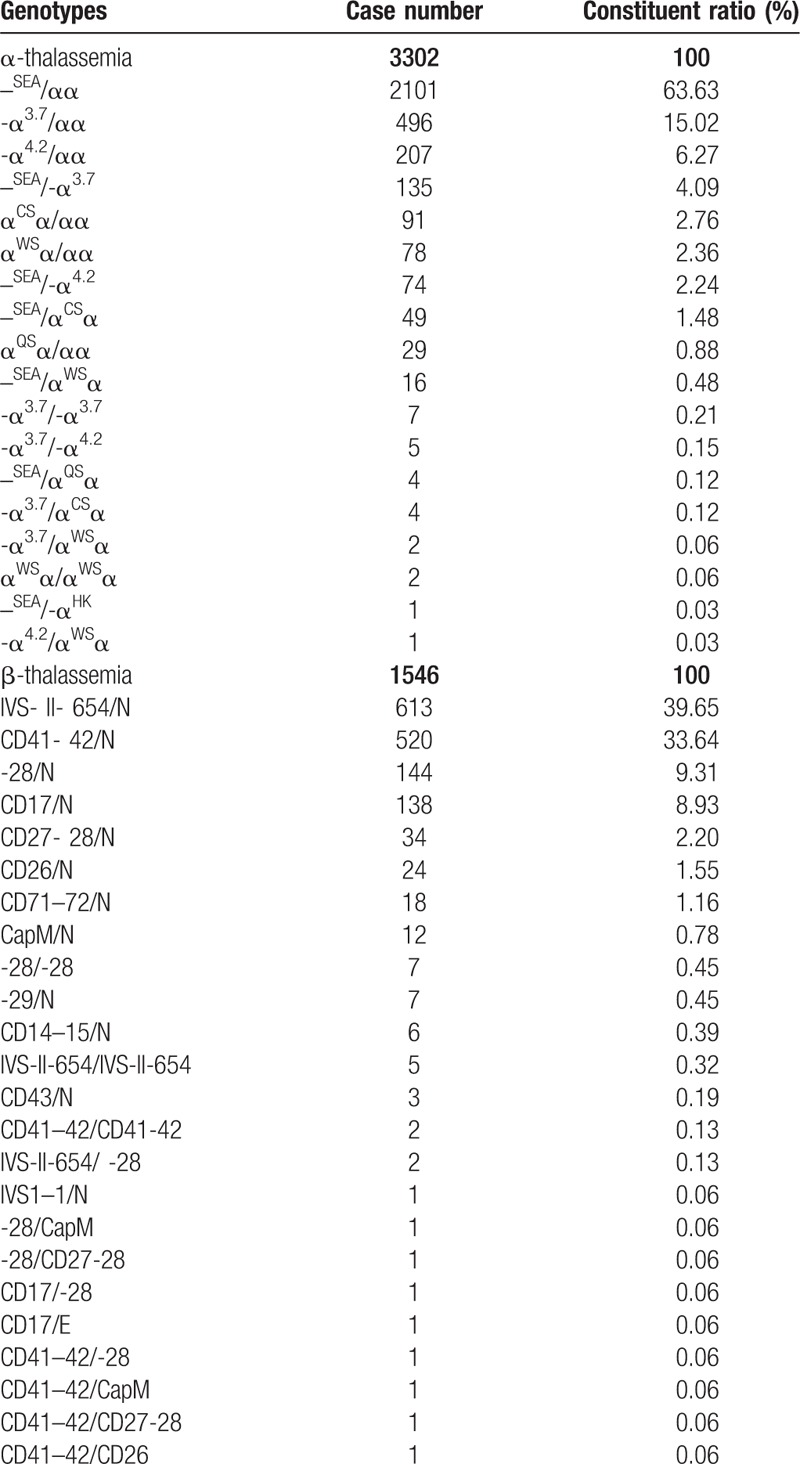
α- and β-thalassaemia genotypes and their distribution among these regions in Hakka.

**Table 4 (Continued):**
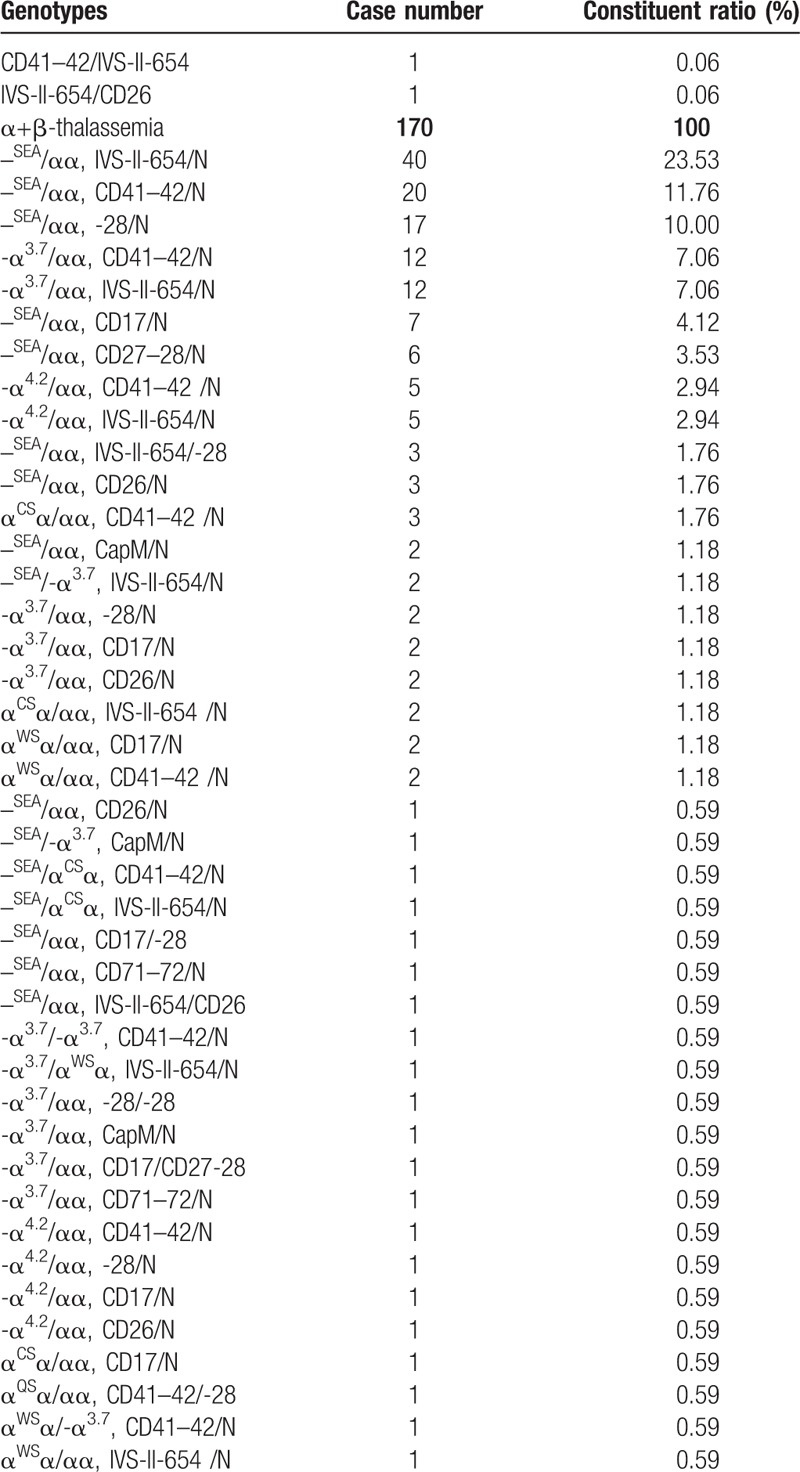
α- and β-thalassaemia genotypes and their distribution among these regions in Hakka.

We found a case with (-α^HK^) deletion by MLPA. In this case, we found the abnormal result when we detected by flow-through hybridization technology on commonly known sites. We analyzed it by MLPA. The result was (--SEA) deletion compound (-α^HK^) deletion.

### 3.3 Molecular prenatal diagnosis of α- and β-thalassemia

**Figure 3:**
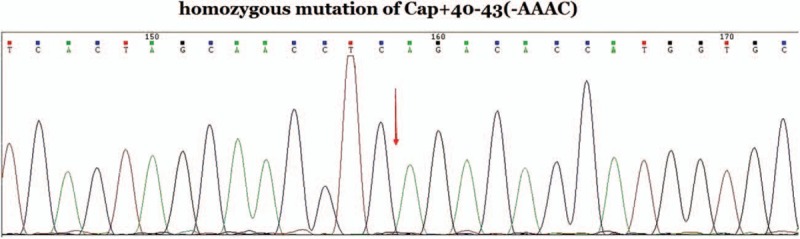
A case with Cap+40–43 (-AAAC) homozygous mutation of β-thalassemia in molecular prenatal diagnosis by Sanger sequencing.

In all Hakka participants, we detected 26 couples carrying the same type of thalassemia. Their fetuses (26 couples’ 27 fetuses) were subjected to prenatal gene diagnosis of thalassemia after informed consent forms have been obtained. We tested 4 CVS samples, 22 amniocentesis fluid samples, and 1 cord blood sample. Five fetuses with Bart's hydrops syndrome, 2 fetuses with β-thalassemia major and 1 with homozygous Cap+40–43 (-AAAC) β-thalassemia mutation (Fig. 3). There is no normal control for Cap+40–43 (-AAAC) mutation in the kit we used, so we detected it using Sanger sequencing. The results of prenatal gene diagnosis in 27 fetuses showed in Table 5.

**Table 5:**
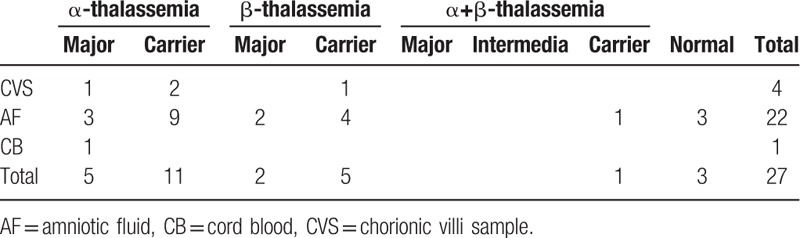
Results of prenatal diagnosis for thalassemia by DNA analysis.

## 4 Discussion

Guangdong province of China suffers a high incidence of thalassemia.^[10,11]^ Meizhou is a mountainous city, located in the northeastern part of Guangdong province, which the vast majority of the permanent residents are Hakka people. The α- and β-thalassaemia prevalence among Hakkas in this region was 34.55%. Compared with previous studies, the higher thalassemia prevalence in Hakka population that shown in our research may be related to the selection of the subjects.^[10–12,21]^ Most of our subjects are hospitalized patients, not random large crowd, which is also a limitation of this study. If we can effectively control of the birth of severe thalassemia in this region, it will produce huge social and economic benefits. The implementation of thalassemia prevention and control requires us first to understand the epidemiology of thalassemia in the region. This study serves this purpose.

Our study showed that --^SEA^/αα was the most common α-thalassemia genotype, followed by -α^3.7^/αα and -α^4.2^/αα. Additionally, it showed that IVS-II-654/N, CD41-42/N, -28/N, CD17/N genotypes accounted for 91.53% of β-thalassemia genotypes. Our results are in line with the results of previous studies in Hakka populations.^[12]^

Meanwhile, the subtle difference of distribution and proportion of the mutant genotypes of thalassemia in those 8 counties of Meizhou are available in our study. The results in α-thalassemia showed that the proportion of the -α^4.2^ deletion of the Hakka people in Jiaoling County was higher than the Hakka people in other counties in Meizhou. On the contrary, the proportions of --SEA deletion and α^WS^α(CD122, CAC→CAG) mutation were relatively lower. The proportion of the α^CS^α(CD142, TAA→CAA) mutation in the Dabu Hakka people was higher than the Hakka people in other areas. For β-thalassemia, our study showed that CD41-42(-TCTT) was the most common mutation in Wuhua and Heyuan populations, followed by IVS-II-654(C→T), -28(A→G), and CD17(A→T).

The mothers of 7 affected fetuses, as mentioned at the results, decided to terminate the pregnancy and were followed up until termination, at the same time tissues were examined and the results were confirmed. Thus, we established prenatal diagnosis can effectively prevent the birth of children with severe thalassemia.

Thalassemia as an endemic disease with high incidence, is general to employ large-scale screening and detection of the carriers, take various measures to prevent and control severe thalassemia birth and safeguard the health of patients to improve the quality of life of the people, because there is no effective treatment method,^[22,23]^ the main means to deal with the prevention of thalassemia. Large data analysis results, public health education among the people of childbearing age, screening, and prenatal diagnosis are the effective means to prevent and control the birth of kids with thalassemia major. This is also the starting point and purpose of our research.

## 5 Conclusion

There were some differences in molecular characterization of thalassemia among Hakka people in different areas of southern China. Our results enriched the related information of thalassemia in the region, which provided valuable basis for the prevention and control of thalassemia. Our study was a retrospective analysis or the detection results related to hospitalized patients, and the results of this study may be limited. A systematic and more large-scale epidemiological survey of the Hakka populations is the way ahead of us.

## 6 Contributions

Pingsen Zhao conceived and designed the experiments; Heming Wu and Pingsen Zhao collected clinical data. Heming Wu and Ruiqiang Weng conducted the laboratory testing. Pingsen Zhao, Heming Wu, and Ruiqiang Weng prepare the manuscript. Pingsen Zhao reviewed the manuscript.

## Acknowledgments

The author would like to thank other colleagues who were not listed in the authorship of Center for Cardiovascular Diseases, Clinical Core Laboratory and Center for Precision Medicine, Meizhou People's Hospital (Huangtang Hospital), Meizhou Hospital Affiliated to Sun Yat-sen University for their helpful comments on the manuscript.

## Author contributions

**Conceptualization:** Pingsen Zhao.

**Data curation:** Pingsen Zhao.

**Formal analysis:** Pingsen Zhao, Heming Wu, Ruiqiang Weng.

**Funding acquisition:** Pingsen Zhao.

**Investigation:** Pingsen Zhao, Heming Wu, Ruiqiang Weng.

**Methodology:** Pingsen Zhao, Heming Wu, Ruiqiang Weng.

**Project administration:** Pingsen Zhao.

**Resources:** Pingsen Zhao, Ruiqiang Weng.

**Software:** Pingsen Zhao, Heming Wu, Ruiqiang Weng.

**Supervision:** Pingsen Zhao.

**Validation:** Pingsen Zhao, Heming Wu, Ruiqiang Weng.

**Visualization:** Pingsen Zhao.

**Writing – original draft:** Heming Wu.

**Writing – review & editing:** Pingsen Zhao.
